# Tracking of Maternal Physical Activity and Sport Participation over 11 Years: Findings from the Czech ELSPAC Study

**DOI:** 10.3390/ijerph19020705

**Published:** 2022-01-09

**Authors:** Mario Kasović, Lovro Štefan, Pavel Piler, Martin Zvonar

**Affiliations:** 1 Department of General and Applied Kinesiology, Faculty of Kinesiology, University of Zagreb, 10 000 Zagreb, Croatia; mario.kasovic@kif.hr; 2 Department of Sport Motorics and Methodology in Kinanthropology, Faculty of Sports Studies, Masaryk University, 62 500 Brno, Czech Republic; 3 Department of Research and Examination (RECETOX), Faculty of Science, Masaryk University, Kotlarska 2, 62 500 Brno, Czech Republic; pavel.piler@recetox.muni.cz (P.P.); zvonar@fsps.muni.cz (M.Z.)

**Keywords:** stability, sport participation, motherhood, longitudinal analysis

## Abstract

*Purpose*: Tracking of physical activity (PA) and sport participation (SP) during motherhood is poorly understood. The purpose of the study was to analyze the extent of tracking of maternal PA and SP. *Methods*: In this investigation, data were collected from the Czech ELSPAC study subsample of 4811 and 2609 women measured postnatally (1991–1992) and after 11 years of follow-up (2002–2003), respectively. The structured questionnaire was used to assess the participation and average weekly time spent in PA, and the frequency of engaging in different sports (running, cycling, strength training, racket sports, swimming, and team sports). Tracking was calculated using generalized estimating equations (GEE) with beta coefficients (β), odds ratios (ORs), and 95% confidence intervals (95% CI). *Results*: Moderately high tracking coefficients were observed for cycling (β = 0.69, 95% 0.67–0.72), strength training (β = 0.59, 95% 0.56–0.63), and weekly time spent in PA (β = 0.53, 95% 0.38–0.66); meanwhile, moderate tracking coefficients were generated for swimming (β = 0.48, 95% 0.44–0.52), team sports (β = 0.44, 95% 0.39–0.48), racket sports (β = 0.44, 95% 0.39–0.48), and running (β = 0.35, 95% 0.30–0.40). Mothers who did not participate in PA at baseline were 81% more likely not to participate in it at follow-up (OR = 1.81, 95% CI 1.53–2.13). *Conclusion*: Cycling- and strength-related activities and weekly PA were tracked moderately-to-moderately high during motherhood. Moreover, the strong tracking of physical inactivity indicates that the detection of this risk factor before pregnancy should be advocated.

## 1. Background

Lack of physical activity (PA) has become a major public health concern worldwide [1]. Although the level of insufficient PA was stable in the last two decades, evidence suggests that global age-standardized prevalence of insufficient PA is 27.5%, with higher levels being predominantly observed in Western and high-income countries [1]. Previous studies have shown that lower levels of PA are consistently associated with weight gain [2], cardiovascular, mental, and metabolic diseases, and all-cause mortality [3].

Despite the positive health benefits of participating in regular PA [4], between 25% and 40% of women in the United States [5] and Australia [6] do not participate in regular PA. Even lower participation in PA has been observed for women in the postpartum period [7]. The prevalence of PA in women is 4% for competitive sport, 25% for moderate-to-heavy activities, and 66% for light activities [7]. It has been documented that women who maintain or increase their PA levels during and after pregnancy may experience better well-being in later life [8].

The promotion of PA is often based on the premise that it has a certain tracking characteristic over time [9]. Tracking characteristic of a single parameter is defined as: (1) “a tendency of individuals to maintain their rank within a certain group over a period of time” [10] and (2) “the ability to predict future observations based on earlier values” [11]. The tracking of PA is especially important for promoting a physically active lifestyle over long periods [9]. To date, several studies have explored the tracking of PA in adult populations [12,13,14,15,16,17,18,19,20,21]. In general, the tracking coefficients for women in these studies ranged between 0.26 and 0.42, indicating low-to-moderate tracking characteristics of PA [9]. The great heterogeneity between the studies may be explained by using self-reported (questionnaires [14,15,16,18,20,21] and interviews [13,17,19]) and objective data to assess PA [12], as well as the follow-up time and sociodemographic characteristics of the study participants [9].

Although a great effort to track women’s PA has been observed [9], the tracking of participating in a specific organized sport activity has been less studied. It has been suggested that engaging in sport may reduce the risk of gestational diabetes mellitus and preterm birth [22]. Studies have shown that the most common leisure activities reported by women include swimming, strength training, running, and cycling, while team and racket sports are less prevalent, due to a high risk of falling and dropping a potential contact [22]. Since PA tends to decline after pregnancy [23], it is necessary to examine how well PA and some specific sport-related activities track into the future. Given the importance of lifelong PA for health, interventions that promote sport-related activities and reduce physical inactivity should be the cornerstones for prevention of non-communicable diseases during motherhood.

Therefore, the purpose of the present study was to analyze the extent of tracking of maternal PA and SP. We hypothesized that both PA and SP would track at least moderately well during the follow-up period of 11 years.

## 2. Materials and Methods

### 2.1. Study Design and Participants

Study data were derived from the Czech part of the ELSPAC study, a prospective birth cohort study carry out in the Czech Republic. Additional details about the study have been published [24]. The Czech part was approved for adherence to ethical guidelines by the Scientific Council of Masaryk University and all participating women provided written informed consent. In this study, a total of 4811 mothers completed first self-reported postnatal questionnaires and were again re-examined 11 years later (*N* = 2609). The procedures performed in this study were anonymous and according to Declaration of Helsinki. The ELSPAC study of the RECETOX department of the Faculty of Science Ethics Committee approved the study.

### 2.2. Variables Used in the Study

The present study used data from questionnaires completed postnatally (x ± sd = 23.4 ± 4 years) and 11 years later (x ± sd = 34.6 ± 0.8 years), designed to elicit information on participating in PA (“yes”/“no”), weekly time spent in PA (during leisure time, open-ended answer, indicated in hours), and the frequency of participating in different sport-related activities (running, cycling, strength training, racket sports, swimming, and team sports). For each sport-related activity, the participants were asked: “How frequently did you participate in a following sport-related activity in the last month?”. Possible answers were: (i) never, (ii) 1x/month, (iii) 1x/week, (iv) 2–3x/week, (v) 4–5x/week, and (vi) 6–7x/week. Covariates in the study included age, body mass index (BMI; calculated from objectively measured height and weight), the first-born child’s gender (a boy vs. a girl), educational level categorized as “low” (primary school), “medium” (secondary school), and “high” (higher education), and self-rated health, ranked from “very poor” to “very good”. Responses “very poor”, “poor”, and “fair” were collapsed into “poor” vs. “good” (“good” and “very good”) self-rated health [24]. In addition, BMI was categorized into “normal weight” (<85th percentile), “overweight” (85th–<95th percentile), and “obesity” (≥95th percentile), according to the International Obesity Task Force (IOTF) [25].

### 2.3. Statistical Analysis

Descriptive statistics at baseline and after a follow-up period of 11 years are presented as mean and standard deviations (SD) for normally distributed variables and median with interquartile range (25th–75th percentile) for not normally distributed variables. Categorical variables are presented as percentages (%). Differences for normally and not normally distributed variables between the two time periods were analyzed using the paired sample *t*-test and the Wilcoxon signed-rank test, while the differences in categorical variables were calculated using cross-tabulation matrices. The tracking of weekly time spent in PA and participating in a different sport-related activity was assessed using generalized estimating equations (GEE). To describe the extent of tracking, we used the tracking coefficient derived from the GEE analysis. The analysis is based on the premise, that the value of the baseline measurement is regressed on the entire longitudinal development of that variable at follow-up. The unique obtained regression (β) coefficient is called the tracking coefficient [26]. This coefficient ranges from 0 to 1, with 1 indicating perfect tracking and 0 indicating no tracking. On the other hand, to evaluate the tracking of participating in PA measured on a “dichotomous” scale (“yes”/“no”), we used odds ratios (ORs) derived from the GEE analysis. To track physical inactivity (a “no” answer at baseline), we used binary regression analysis in GEE, to determine the odds of remaining in a physically “inactive” group at follow-up. Two-sided *p*-values were used, and significance was set at α < 0.05. All the analyses were calculated in Statistical Packages for Social Sciences v.23 (SPSS, Chicago, IL, USA).

## 3. Results

Descriptive statistics of the study participants are presented in Table 1. Table 1 reveals an increase in mother’s weight (mean difference = 4.0 kg, 95% CI 3.9–4.1 kg, *p* < 0.001) and BMI (mean difference = 1.4 kg/m^2^, 95% CI 1.2–1.6, *p* < 0.001) and a decrease in average weekly time spent in PA (*Z* = −16.0, *p* < 0.001). Additionally, the prevalence of mothers who participated in regular PA at baseline dropped by 18% at follow-up (*p* < 0.001) and the proportion of mothers reporting “good” self-rated health decreased by 4.3% (*p* < 0.001). In addition, the prevalence of “overweight” and “obesity” increased from 9.5% and 2.3% to 18.7% and 6.5% at follow-up (*p* < 0.001).

**Figure 1:**
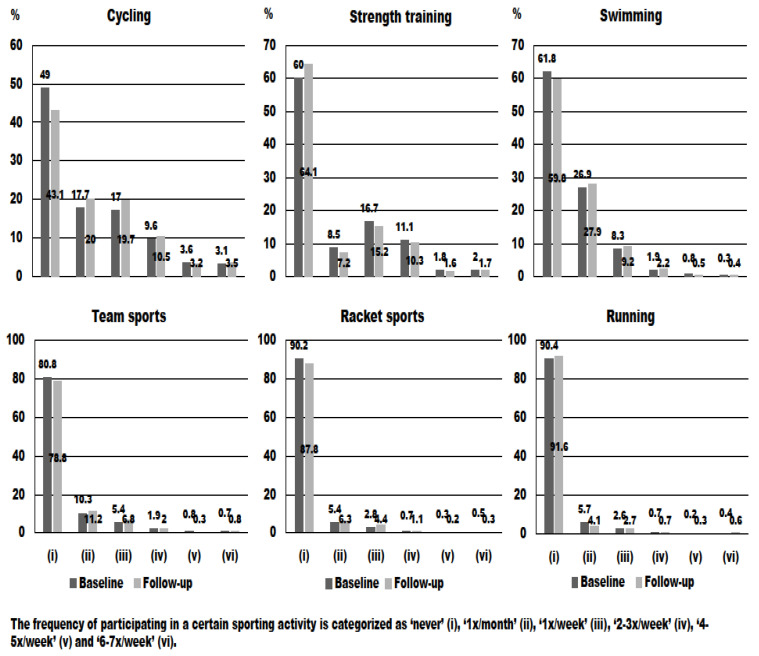
The frequency of participating in different sport-related activities at baseline and follow-up.

Figure 1 presents the frequency of participating in different sport-related activities at baseline and follow-up. As shown in Figure 1, most mothers “never” participated in a sport activity, i.e., the highest percentages for running, racket sports, and team sports (>80%) were observed. On the other hand, between 40% and 50% of the study participants participated in cycling, strength training, and swimming. Additionally, only a very small proportion of participants reported participating in sport activities from 2–3x/week to 6–7x/week. When observing PA, 47.8% of mother who were physically active at baseline remained physically active at follow-up, while 44.0% of mothers physically inactive at baseline remained being physically inactive at follow-up. On the other hand, 35.5% of mothers who were physically active at baseline stopped being physically active at follow-up and 35.4% of them who were physically inactive at baseline were physically active at follow-up. Finally, 81.1% of mothers who engaged in PA for ≥150 min/week remained in the same category at follow-up.

**Figure 2:**
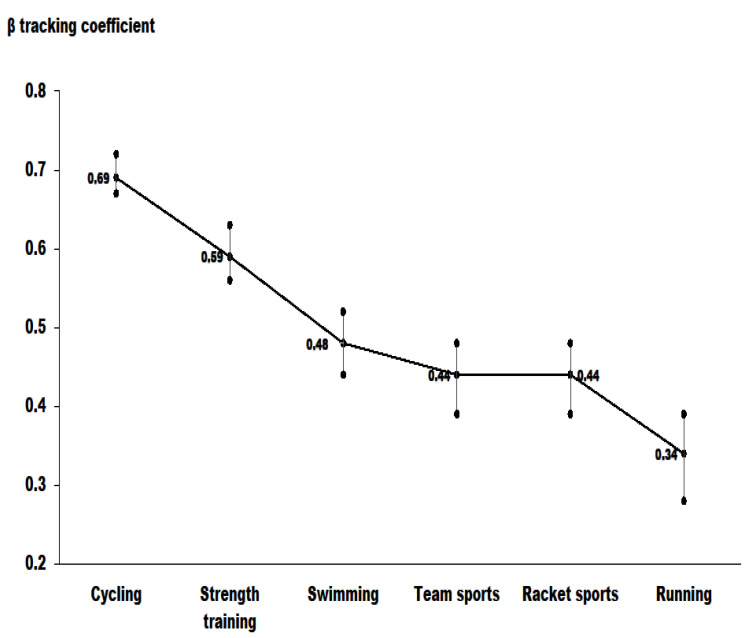
Tracking coefficients (β) with 95% CI for each sport-related activity.

The results for tracking coefficients of each sport activity are presented in Figure 2. After adjusting for maternal age, body mass index, education, self-rated health, and child’s gender, moderately high tracking coefficients were observed for cycling and strength training, while swimming, team sports, racket sports, and running tracked moderately well over 11 years of follow-up. Of note is that the average weekly time spent in PA also showed a moderately high tracking characteristic (β = 0.53, 95% 0.38–0.66). Tracking of participation in PA showed that mother who did not participate in PA at baseline were 81% more likely not to participate in PA at follow-up (OR = 1.81, 95% CI 1.53–2.13, *p* < 0.001). Note that those mothers who were active in a given sport at baseline were 2.10 times (OR = 2.10, 95% CI 1.76–2.52, *p* < 0.001) more likely to be physically active at follow-up.

## 4. Discussion

The main purpose of the present study was to analyze the extent of tracking of maternal PA and SP. Our main findings are: (a) weekly time spent in PA tracked moderately high; (b) the participation in cycling and strength training showed moderately high tracking, while swimming, team sports, and racket sports tracked lower, which could be interpreted as moderate; (c) women who did not participate in PA at baseline were 81% more likely not to participate in PA at follow-up.

The tracking of weekly time spent in PA and participating in different sport has been defined as moderate–moderately high in this study, which is larger than in the most studies that investigated the tracking characteristics of PA in women [12,13,14,15,16,17,18,19,20,21]. The tracking coefficients of PA in these studies ranged between 0.12 and 0.61, indicating low to moderately high tracking. Specifically, an Australian study using pedometers to assess PA reported low to moderately high tracking of 0.20 to 0.61 in a women cohort [12]. In three other studies, the tracking coefficients varied from 0.35 to 0.42 [13,14,15]. Somewhat lower tracking coefficients of PA were observed in one Finnish (*r* = 0.31) and one American study (*r* = 0.30) [16,19]. Higher tracking observed in our study may be explained by using only a single-item question to assess the weekly time spent in PA, while other studies used more detailed questionnaires. Additionally, higher tracking coefficients for different sporting activities can be associated with a better recall, since these activities are often organized and determined by intensity, frequency, and duration. It has been suggested that organized sport performed during childhood and adolescence is a strong predictor of future PA in both prospective and retrospective studies [27,28,29]. Thus, earlier experience in sports make it easier to maintain PA or start it again [9].

We also observed that some aerobic and muscle-strengthening activities, such as cycling and participating in fitness groups, tracked moderately high into motherhood. The tracking of the same type of PA has been little studied. For example, adolescents who participated in endurance type of sport (cycling) had a higher probability of participating in the same kind of activities 17 years later [30,31]. Similar associations were found for swimming [32]. Previous evidence has suggested that the most common leisure activities reported by women include weightlifting, aerobics classes, swimming, and cycling. The guidelines from the 2002 American College of Obstetrics and Gynecology (ACOG) recommend that women participate in recreational activities with a lower risk of injuries and try to avoid a high potential for contact activities, including vigorous team and racket sports [33]. Therefore, it is not surprising that we obtained lower tracking coefficients for the participation in these activities. Additionally, higher tracking of the aforementioned activities could be explained by the changes in opportunities for PA in women, especially related to indoor cycling and strength activities [9].

### Strengths and Limitations

This study has several strengths. The findings presented in this paper may be used for comparative studies. The Central European population of children born in the early 1990s grew up under specific socioeconomic and cultural conditions, which were very different from those in the Western part of Europe [34]. A population-based longitudinal design allows us to establish a certain causality between the time points, and to observe changes in a given variable. We also analyzed several different sporting activities, which allowed us to see that cycling and strength training activities tracked moderately high during the period of 11 years. Finally, we adjusted for several potential covariates, which could influence the tracking characteristics. Nevertheless, regardless of the significance of the present results, several limitations of the current study should be noted. First, the limitation of the present study was the use of questionnaires to assess PA, which typically leads to an overestimation and a larger measurement error of PA [35]. However, studies have shown that the tracking using self-reported and that using objective methods are quite similar [9]. The reason may be that, although objective methods measure PA more accurately, their ability to capture a sufficient frequency of individual’s activities during the day is lower [9]. Second, biological samples (biochemical and physiological parameters) of women were not assessed at the baseline. Third, the tracking coefficients were derived using only two data points. If we had more frequent data points (for example one per year), the magnitude might have been different.

## 5. Conclusions

The main purpose of the present study was to analyze the extent of tracking of maternal PA and participation in various sporting activities, when controlling for mother’s age, BMI, educational level, child’s gender, and self-rated health. We hypothesized that the tracking of PA and each sport activity would be at least moderate. For cycling and strength training activities, the tracking coefficients were moderately high, while for others, a moderate tracking was observed. Therefore, we can conclude that the hypothesis was confirmed. In addition, women with no reported participation in PA at baseline were 1.81 times more likely not to participate in PA at follow-up.

## 6. Perspective

Moderately high tracking of cycling and muscle-strengthening activities should be an avenue for promoting healthy lifestyles. Based on our findings, women during motherhood most frequently participate in the aforementioned activities, followed by swimming, racket and team sports, and running. Some studies show that the level of PA after pregnancy significantly drops, due to the major transitions in the course of life, i.e., from singlehood to marriage and having children, in addition to many life changes, have a greater influence on the PA of women, compared with men [36]. Moreover, interventions that use activity trackers and smartphone apps to increase PA in both children and parents are strongly recommended [37].

## Figures and Tables

**Table 1:** Descriptive statistics of the study participants at baseline and follow-up.

	Baseline			Follow-Up		
Numerical Variables	*N*	Mean	SD	*N*	Mean	SD
Age (years)	4612	23.4	0.5	2480	34.6 **	0.8
Height (cm)	4544	166.3	6.0	2485	166.5	6.0
Weight (kg)	4758	61.2	12.6	2482	65.2 **	11.8
Body mass index (kg/m^2^)	4517	22.1	3.3	2471	23.5 **	4.0
Weekly physical activity (h) *	1920	7.0	4.0–13.0	1156	2.0 **	1.0–3.0
Categorical variables	N	%	95% CI		%	95% CI
Physical activity						
Yes	2392	48.6	46.8–50.4	1085	30.6 **	28.9–32.3
No	2328	51.4	49.6–53.2	2577	69.4 **	67.7–71.1
Physical activity						
<150 min/week	463	24.1	22.8–25.9	512	44.3 **	41.9–46.8
≥150 min/week	1457	75.9	74.1–77.2	644	55.7 **	54.1–57.4
Child’s gender						
A boy	2400	49.9	47.4–52.4	/	/	/
A girl	2411	50.1	47.6–52.6	/	/	/
Educational level						
Low	505	10.6	9.7–11.4	/	/	/
Medium	3209	67.1	65.8–68.4	/	/	/
High	1070	22.4	21.2–23.5	/	/	/
Self-rated health						
Poor	470	8.3	7.0–9.7	250	12.6 **	11.2–14.2
Good	4298	91.7	90.3–93.0	1738	87.4 **	85.8–88.8
Body mass index categories						
Normal weight	3856	88.2	86.8–89.7	1808	74.8 **	72.9–76.8
Overweight	489	9.5	8.1–10.7	461	18.7 **	16.8–20.5
Obese	144	2.3	1.6–2.9	167	6.5 **	5.3–7.6

* denotes using median and interquartile range (25th–75th percentile). ** denotes a significant change from baseline to follow-up.

## Data Availability

The datasets analyzed during the current study are available on reasonable request through the website of the Czech ELSPAC project: http://www.elspac.cz/index-en.php (accessed on 9 December 2021).
